# Antifungal Efficacy of Antimicrobial Peptide Octominin II against *Candida albicans*

**DOI:** 10.3390/ijms241814053

**Published:** 2023-09-13

**Authors:** J. N. C. Jayasinghe, Ilson Whang, Mahanama De Zoysa

**Affiliations:** 1 College of Veterinary Medicine and Research Institute of Veterinary Medicine, Chungnam National University, Daejeon 34134, Republic of Korea; nirmanijay@o.cnu.ac.kr; 2 National Marine Biodiversity Institute of Korea (MABIK), Janghang-eup 33662, Republic of Korea

**Keywords:** *Candida albicans*, *Octopus minor*, Octominin II, antifungal activity, toxicity

## Abstract

Most clinically isolated *Candida albicans* strains are drug-resistant, emphasizing the urgent need to discover alternative therapies. In this study, the previously characterized Octominin was modified into a shorter peptide with an 18 amino acid sequence (^1^GWLIRGAIHAGKAIHGLI^18^) and named Octominin II. The secondary structure of Octominin II is a random coil with a helical turn and a positive charge (+2.46) with a hydrophobic ratio of 0.46. Octominin II inhibited *C. albicans*, *C. auris*, and *C. glabrata* with minimum inhibitory and fungicidal concentrations against *C. albicans* of 80 and 120 µg/mL, respectively. Field emission scanning electron microscopy confirmed that Octominin II treatment caused ultra-structural changes in *C. albicans* cells. Furthermore, membrane permeability results for the fluorescent indicator propidium iodide revealed modifications in cell wall integrity in Octominin II-treated *C. albicans*. Octominin II treatment increases the production of reactive oxygen species (ROS) in *C. albicans*. Gene expression studies revealed that Octominin II suppresses virulence genes of *C. albicans* such as *CDR1*, *TUP1*, *AGE3*, *GSC1*, *SAP2*, and *SAP9*. In addition, a nucleic acid binding assay revealed that Octominin II degraded genomic DNA and total RNA in a concentration-dependent manner. Additionally, Octominin II inhibited and eradicated *C. albicans* biofilm formation. Octominin II showed relatively less cytotoxicity on raw 264.7 cells (0–200 µg/mL) and hemolysis activity on murine erythrocytes (6.25–100 µg/mL). In vivo studies confirmed that Octominin II reduced the pathogenicity of *C. albicans*. Overall, the data suggests that Octominin II inhibits *C. albicans* by employing different modes of action and can be a promising candidate for controlling multidrug-resistant *Candida* infections.

## 1. Introduction

*Candida* spp. are opportunistic pathogens in the commensal flora, and their infections have increased over past decades, causing a hassle to the healthcare system [1,2]. *Candida* spp. cause superficial infections known as candidiasis and systemic infections known as candidemia [3]. Among the species of the *Candida* family, *Candida albicans* has been identified as the prime pathogen. The pathogenicity of *Candida* is influenced by different factors, such as its ability to thrive under extreme and different environmental conditions, which are supported by virulence factors, including its ability to form biofilms on various anatomical surfaces and drug resistance [2,3]. Candidemia is the fourth cause of mortality related to nosocomial infections, aided by an increasing immune-compromised population in the world [3,4,5]. Current anticandidal drugs against candidiasis, such as azoles (fluconazole), polyenes (amphotericin), and echinocandins (micafungin), show less efficacy due to the development of multidrug resistance. Hence, it has been predicted that the mortality rate of *Candida* infections will continue to increase [1,6,7]. *Candida* has shown diverse mechanisms to escape the effects of antibiotics, such as drug target alteration, drug target overexpression, and efflux pump overexpression [6]. Considering the gravity of the crisis caused by this pathogen, new drugs are urgently required.

Antimicrobial peptides (AMPs) have recently gained attention owing to their potential use as antibiotics to mitigate antimicrobial resistance. AMPs are small proteins of ≤50 amino acids in length that can be found naturally in all living organisms, including mammals, amphibians, insects, microorganisms, aquatic organisms, plants, and also in cancerous cells. These peptides are called ‘host defense peptides’. AMPs mostly show cationic characteristics, wherein the net charge ranges from +2 to 11 with a higher proportion of hydrophobic residues than hydrophilic residues [8,9]. AMPs vary in their secondary structures from alpha helices, β sheets, αβ sheets, and random coils [9]. Since AMPs carry extremely diverse antimicrobial mechanisms and attach to multiple low-affinity points, they prevent and slow down the occurrence and evolution of resistance [8,10]. AMPs from different sources, both natural and synthetic, have been studied for their effectiveness against various microorganisms, including bacteria, fungi, and viruses [11].

Although AMPs are readily found in natural sources, their use in therapeutics is not feasible. Researchers have focused more on synthetic peptides because they can be easily designed, modified, and synthesized in large quantities with high purity, and the activity of AMPs can be predetermined using bioinformatic tools coupled with machine learning [12,13]. Promising AMPs have been developed using defense protein sequences from various organisms [14,15,16,17,18,19]. Our research group has reported a 23 amino acid length AMP, named Octominin. It has shown multiple antimicrobial activities against *Candida albicans* [11], *Streptococcus parauberis* [20], and *Acinetobacter baumanii* [21], as well as anti-inflammatory effects [22].

In this study, we modified the original Octominin sequence into a shorter fragment (18 amino acids) and named it Octominin II. The antifungal activity of Octominin II was determined by evaluating the minimum inhibitory/fungicidal concentration (MIC/MFC), ultrastructural changes, membrane permeability, transcription, and antibiofilm activities. Moreover, the internal cellular targets were studied with respect to the DNA- and RNA-binding ability of Octominin II. Additionally, the toxicity/safety level of Octominin II was determined in vitro (RAW 264.7 cells) and in vivo (zebrafish embryos and larvae) and hemolysis activity (murine red blood cells). Finally, an in vivo study was performed to investigate the therapeutic efficacy of Octominin II in adult zebrafish challenged with *C. albicans*. The overall results of this study confirmed that Octominin II could be a potential anticandidal agent for controlling *Candida* spp. effectively.

## 2. Results

### 2.1. Physiochemical Characteristics of Octominin II

**Figure 1:**
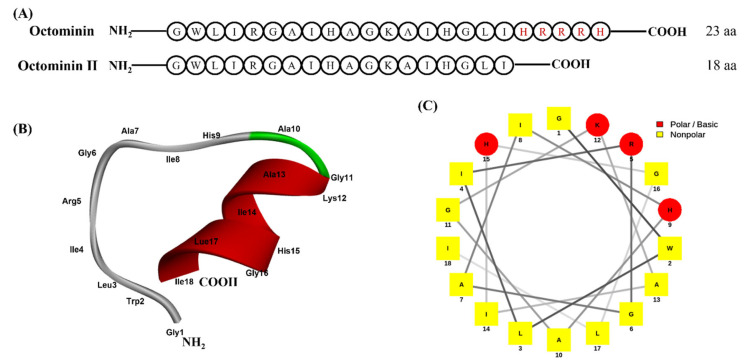
Amino acid sequence comparison-predicted secondary structure and the helical wheel projection of Octominin II. (**A**) The amino acid sequence of Octominin and Octominin II. Positively charged amino acids are in red. (**B**) Three-dimensional structure of Octominin II. Predicted as a random coil with a turn is shown from Ala10 to Leu17 (turns in green and unstructured in grey). (**C**) The helical wheel projection of Octominin II shows polar (red) and non-polar residues (yellow).

Octominin II was derived from a previously characterized Octominin used as a template sequence by deleting 5 AAs from its C-terminus. The amino acid sequences of Octominin and Octominin II are indicated in Figure 1A. The molecular weight of synthesized Octominin II was 1833.5 Da and it had 98.6% purity (Supplementary Figure S1). The physicochemical properties of Octominin II were determined using the peptides package in R software version 1.2.2 (Table 1). The most important physiological parameters affecting the antimicrobial activity of a peptide include its net charge and hydrophobicity. Octominin II is a cationic peptide with a +2.46 charge and carries arginine, lysine, and histidine as polar residues. Octominin II carries comparatively higher numbers of non-polar residues such as isoleucine, leucine, and glycine, which explains its lower positive charge. The hydrophobicity index of Octominin II was 0.46, which is a moderate value. The moderate hydrophobicity index explains the low cytotoxicity and hemolytic effects described in later sections of this study. The isoelectric point of Octominin II is 11.66, which is also a moderate value that prevents the peptide from aggregating in the solution. The predicted secondary structure of Octominin II revealed a random coil with a helical turn from Gly11 to Leu17 (Figure 1B). The helical wheel projection of Octominin II showed a hydrophilic face containing polar-charged amino acids, such as lysine and arginine (Figure 1C).

### 2.2. Anticandidal and pH Dependent Activity of Octominin II against Candida *spp*.

**Figure 2:**
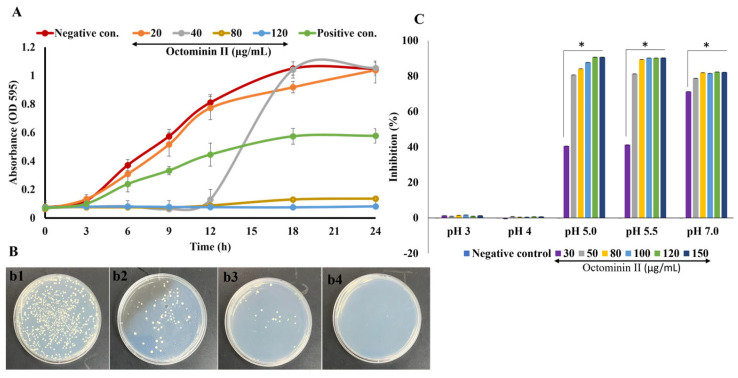
Time-kill kinetics and pH-dependent activity of Octominin II on *C. albicans*. (**A**) Time-kill kinetics of Octominin II. *C. albicans* growth inhibition was assessed after treating with Octominin II (0.25 MIC; 20 µg/mL, 0.5 MIC; 40 µg/mL, MIC; 80 µg/mL and MFC; 120 µg/mL with the positive control (fluconazole; 280 µg/mL)). The growth of *C. albicans* was evaluated at 3 h intervals by measuring the optical density at 595 nm. The error bars indicate the means of standard deviation where *n* = 3. (**B**) Octominin II-treated *C. albicans* was plated at 24 h post-treatment ((**b1**); negative control, (**b2**–**b4**); Octominin II 40, 80, and 120 µg/mL, respectively). (**C**) pH-dependent activity of Octominin II against *C. albicans*. Inhibitory activity of Octominin II was assessed at different pH values and compared to the non-treated negative control. (*n* = 3, *: significant inhibition; *p* < 0.001 compared to the negative control).

The anticandidal activity of Octominin II was assessed against *C. albicans*, *C. auris*, and *C. glabrata*. Octominin II was found to exert species-dependent antifungal activity, based on the different MIC and MFC values obtained for each species. The following were the MIC and MFC for *C. auris* (160 and 200 µg/mL, respectively), *C. albicans* (80 and 120 µg/mL, respectively), and *C. glabrata* (55 and 100 µg/mL, respectively). In addition, fluconazole (positive control)-treated *C. albicans* showed the highest MIC (280 µg/mL). Among them, *C. albicans* was used for further experiments since it is the most prominent *Candida* spp. with a global public health priority. Time-kill kinetics assay was used to determine the growth inhibitory profile of Octominin II at different concentrations (20, 40, 80, and 120 µg/mL (Figure 2A)). The concentration-dependent inhibitory activity of Octominin II was pronounced, with stronger inhibitory activity than that of fluconazole at 280 µg/mL (Figure 2A,B). To investigate the effect of pH on the antifungal activity of Octominin II, we prepared culture media at different pH values (3.0, 4.0, 5.0, 5.5, and 7.0) and compared the inhibition percentages. The anticandidal activity of Octominin II was pH-dependent; however, *C. albicans* growth was not observed at pH 3 and 4 because of the highly acidic conditions in the media (Figure 2C). At pH 7.0, stronger anticandidal activity was observed at the lowest concentration of Octominin II (30 µg/mL).

### 2.3. Ultrastructural Changes in C. albicans Caused by Octominin II

**Figure 3:**
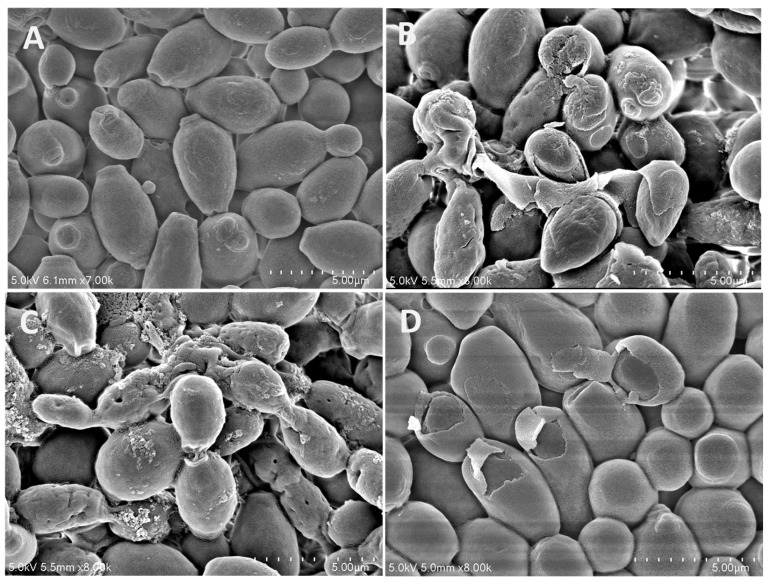
Effect of Octominin II on morphological and structural changes in *C. albicans* assessed by field emission scanning electron microscopy (FE-SEM). *C. albicans*, (**A**); untreated, (**B**); treated with Octominin II (80 µg/mL), (**C**); treated with Octominin II (120 µg/mL), (**D**); treated with fluconazole (280 µg/mL).

Octominin II-treated *C. albicans* (MIC, 80 µg/mL; MFC, 120 µg/mL) were observed using field-emission scanning electron microscopy (FE-SEM) to identify possible ultrastructural changes (Figure 3). The FE-SEM analysis showed that the Octominin II-treated *C. albicans* had defective and altered cell surfaces (Figure 3B,C), whereas untreated cells in the negative control group showed undamaged and smooth cell surfaces without any defects (Figure 3A). Damaged *C. albicans* showed pores and wrinkles on the cell surface, which were also observed in MFC-treated samples. The MFC-treated *C. albicans* had more deformed cells with pores and wrinkles than the MIC-treated samples. However, both the MIC and MFC of Octominin II-treated *C. albicans* caused ultrastructural damage when compared to fluconazole (Figure 3D).

### 2.4. Effect of Octominin II on Cell Membrane Permeability of C. albicans

**Figure 4:**
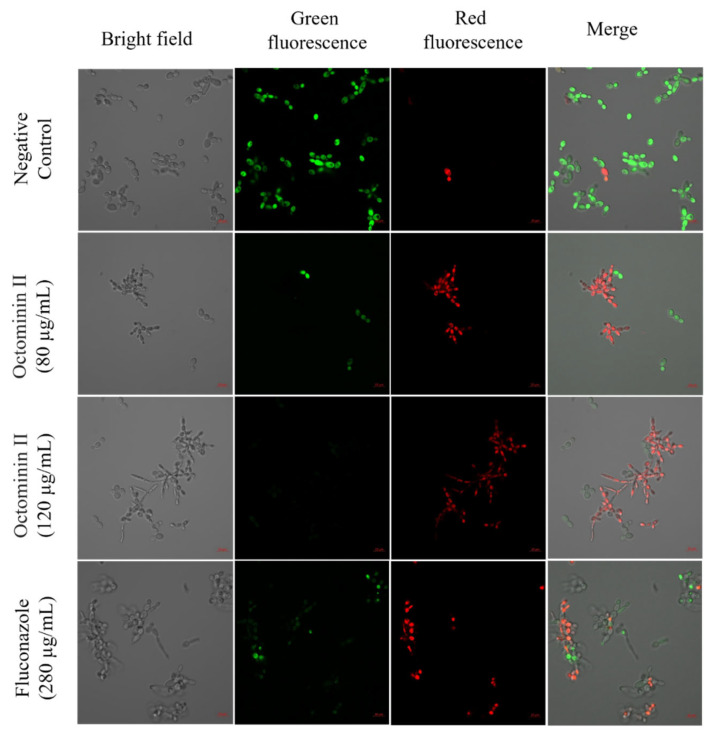
Effect of Octominin II on the membrane permeability of *C. albicans*. PBS-treated as negative control, Octominin II-treated (MIC; 80 µg/mL and MFC; 120 µg/mL) and fluconazole (280 µg/mL) as a positive control. *C. albicans* was incubated at 37 °C for 10 h and stained with PI and FDA. Cells were visualized using CLSM. Red fluorescence (PI: dead cells) and green fluorescence (FDA: live cells) at excitation wavelengths of 535 and 617 nm, respectively, and emission wavelengths of 488 and 535 nm, respectively. Scale bar; 10 µm.

Propidium Iodide (PI)/Fluorescein Diacetate (FDA) assay was performed to identify live and dead cells after Octominin II treatment via fluorescence-based staining. Red fluorescence indicates dead cells by staining the nuclei, and green fluorescence is emitted in FDA in viable cells via metabolism to fluorescein. The results of the PI/FDA analysis showed that cells treated with 120 µg/mL (MFC) and 80 µg/mL (MIC) Octominin II had the highest levels of red fluorescence signals when compared to the negative control (Figure 4). This indicated a possible change in cell permeability and integrity, which may lead to fungal cell death upon Octominin II treatment. Moreover, Octominin II-treated *C. albicans* cells showed weaker green fluorescence signals when compared to the highest green fluorescence signal observed in the negative control. Moreover, Octominin II-treated *C. albicans* cells showed higher red fluorescence and fewer green fluorescence signals when compared to the positive control, fluconazole (280 µg/mL).

### 2.5. Elevated Intracellular Oxidative Stress upon Treatment with Octominin II

**Figure 5:**
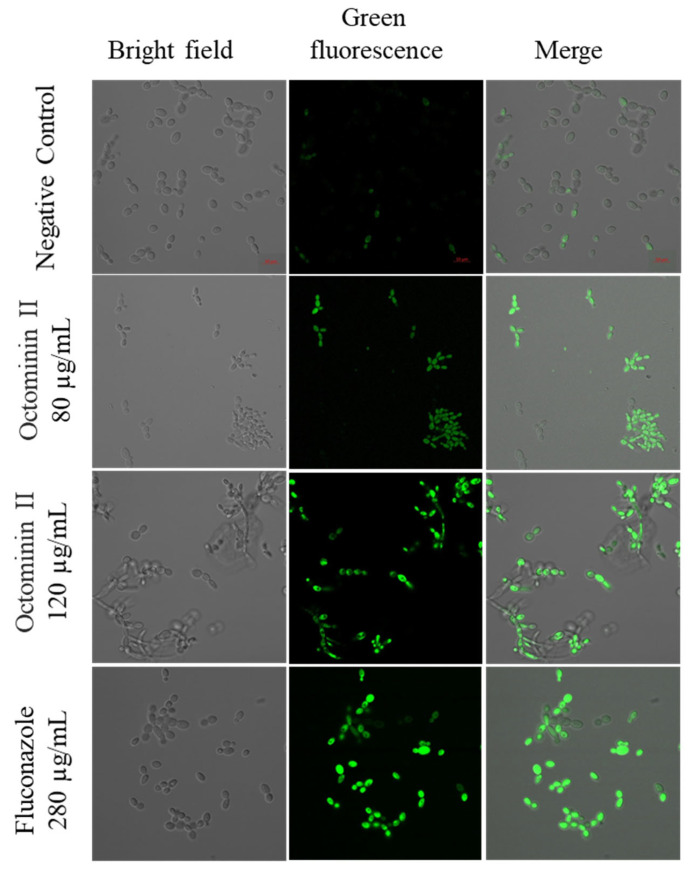
Effect of Octominin II on ROS production in *C. albicans*. CLSM-merged and fluorescence images representing ROS production in *C. albicans* with PBS-treated, Octominin II-treated (MIC;80 µg/mL, MFC;120 µg/mL) and fluconazole (280 µg/mL) as positive control. *C. albicans* were treated with the above-mentioned treatments and incubated for 10 h and stained with H_2_DCFDA. Cells were visualized using CLSM. Green fluorescence was observed at excitation and emission wavelengths of 488 and 535 nm, respectively. Scale bar; 10 µm.

Reactive oxygen species (ROS) include highly reactive superoxide, such as superoxide O_2_^−1^, hydroxyl radical OH^−1^, and hydrogen peroxide (H_2_O_2_), which damage DNA and RNA, elevating oxidative stress in the cell. ROS levels were measured to determine whether the inhibitory activity of Octominin II was related to endogenous oxidative stress caused by ROS production. *C. albicans* treated with 80 µg/mL (MIC) and 120 µg/mL (MFC) of Octominin II emitted a higher level of green fluorescence than the positive control (fluconazole; 280 µg/mL), whereas the negative control exhibited less green fluorescence (Figure 5). This suggests that Octominin II induces ROS and elevates oxidative stress in *C. albicans*, which inhibits cell growth, leading to cell death, and impairs cellular activity by damaging DNA, RNA, proteins, and lipids.

### 2.6. Interaction of Octominin II with Genomic DNA of C. albicans

**Figure 6:**
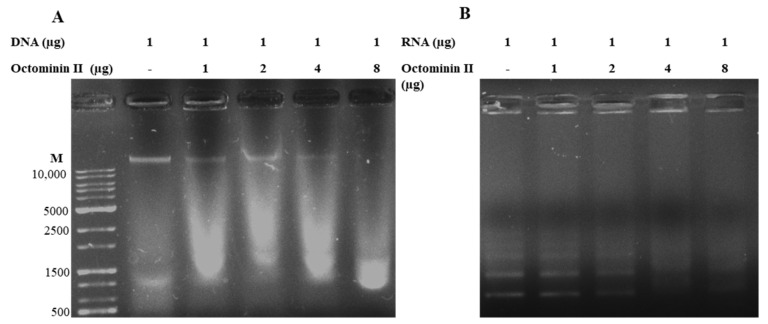
DNA and RNA binding activity of Octominin II. Genomic DNA (200 ng) and RNA (100 ng) isolated from *C. albicans* were treated with Octominin II in varying ratios as: 1:0, 1:1, 1:2, 1:4, and 1:8. (*w*/*w*). Octominin II and DNA/RNA were incubated at 37 °C for 1 h and then loaded (10 μL) into agarose gel (0.8%), and electrophoresis was performed. Interaction between Octominin II and *C. albicans* genomic DNA or RNA was assessed by observing the migration pattern of genomic DNA. (**A**); DNA mobility shift assay, (**B**); RNA mobility shift assay. M: DNA marker.

DNA and RNA mobility shift assays were performed to investigate whether Octominin II is involved in DNA/RNA binding in *C. albicans*. The DNA mobility pattern was observed by agarose gel electrophoresis, as shown in Figure 6. Gel image observations revealed that the intensity of the genomic DNA band decreased, indicating a smear with an increasing ratio of Octominin II (1:1, 1:2, 1:4, and 1:8). It can be suggested that Octominin II may bind to genomic DNA, thereby destroying it, suggesting the suppression of transcriptional regulation of genes associated with cell proliferation and other cellular functions (Figure 6A). A similar pattern was observed in the RNA mobility shift assay, wherein RNA was degraded in a concentration-dependent manner (Figure 6B). Altogether, this DNA- and RNA-damaging effect of Octominin II may disturb transcription in *C. albicans* cells, which may influence the molecular-level survival mechanisms, leading to cell death.

### 2.7. Antibiofilm Activity of Octominin II

**Figure 7:**
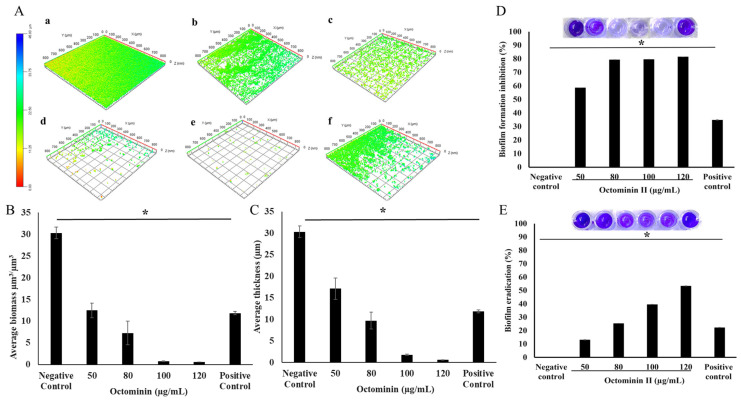
Anti-biofilm activity of Octominin II against *C. albicans*. (**A**) 3D structure of the formed biofilms upon Octominin II treatment. Octominin II: (**a**); 0, (**b**); 50, (**c**); 80, (**d**); 100, and (**e**); 120 µg/mL and (**f**); Positive control (fluconazole 280 µg/mL). (**B**); Biofilm biomass (µm^3^/µm^2^), (**C**); Biofilm thickness (µm). For biofilm formation inhibition, and eradication assays, *C. albicans* planktonic culture and the mature biofilm were treated with varying concentrations of Octominin II (50, MIC; 80, 100, and MFC; 120 µg/mL), and then the formed/remaining biofilm biomass was assessed via crystal violet staining. (**D**) Biofilm formation inhibition effect of Octominin II. (**E**) Biofilm eradication assay. *: *p* < 0.001 compared to the negative control. (*n* = 3).

The antibiofilm activity of Octominin II against *C. albicans* biofilms was assessed using confocal laser scanning microscopy to observe the 3D structure of the *C. albicans* biofilms after Octominin II treatment (0, 50, 80, 100, and 120 µg/mL) and the positive control, fluconazole 280 µg/mL (Figure 7A). Images obtained from confocal laser scanning microscopy (CLSM) were analyzed using the COMSTAT 1 software for quantitative analysis. Negative control (untreated) showed a well-grown biofilm with higher biomass (µm^3^/µm^3^) and thickness when compared to Octominin II treated samples, as indicated in the graphs (Figure 7B,C). In the Octominin II-treated samples, cells were dispersed and had lower biomass and thickness, which was also concentration-dependent. The inhibition of biofilm formation and eradication ability of Octominin II were assessed by evaluating the formed biofilm biomass (%) upon Octominin II treatment when compared to the untreated negative control. The results showed that Octominin II has a concentration-dependent inhibitory activity, which is consistent with the results of the CLSM study (Figure 7D). The biofilm eradication ability of Octominin II was assessed by comparing the remaining biofilm biomass with that of the untreated negative control upon Octominin II treatment. Octominin II showed the highest concentration-dependent biofilm eradication at 120 µg/mL, whereas the lowest was observed at 50 µg/mL (Figure 7E). However, this indicates that a higher concentration of Octominin II is required for biofilm eradication when compared to the inhibition of biofilm formation because mature biofilms have several drug resistance and survival mechanisms.

### 2.8. Effect of Octominin II on the Expression Pattern of Selected Virulence Genes of C. albicans

**Figure 8:**
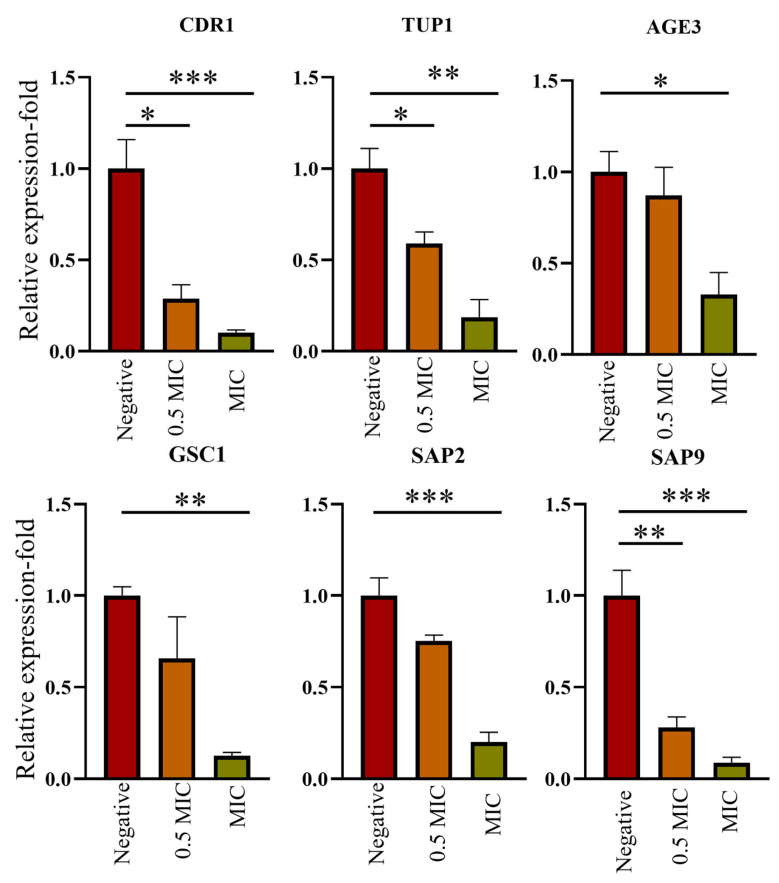
Transcriptional responses of genes of *C. albicans* treated with Octominin II. Multidrug resistance (CDR1), biofilm filamentation, and hyphal growth (*TUP1*, *GSC1*, *AGE3*, *SAP2*, and *SAP9*) were tested. Relative mRNA expression fold of Octominin II-treated at MIC (80 µg/mL) to *C. albicans* normalized with that of PBS-treated control group to compare the fold values. Statistical significance * *p* < 0.05, ** *p* < 0.01, *** *p* < 0.001 compared to the negative control group and presented relative fold values with mean ± SD (*n* = 3).

qRT-PCR was performed to evaluate the transcriptional response of the genes related to virulence of *C. albicans* (Figure 8). CDR1 was significantly suppressed (0.5 MIC; 0.28, MIC; 0.10), supporting drug resistance, a major virulence factor in *C. albicans* that plays a role in pathogenicity. Genes that assist the filamentation and hyphal growth of *Candida* spp. such as *GSC1* (0.5 MIC; 0.65, MIC; 0.12), *AGE3* (0.5 MIC; 0.83, MIC; 0.33), and *TUP1* (0.5 MIC; 0.59, MIC; 0.33) were also downregulated. Secreted aspartic proteases (Saps) *SAP2* (0.5 MIC; 0.75, MIC; 0.20) and *SAP9* (0.5 MIC; 0.28, MIC; 0.09) genes play a key role in nutrient acquisition and virulence by promoting biofilm growth. In summary, all virulence-related genes that were tested were suppressed in 0.5 MIC (40 µg/mL) and MIC of Octominin II (80 µg/mL).

### 2.9. In Vivo and In Vitro Cytotoxicity and Hemolysis Activity of Octominin II

**Figure 9:**
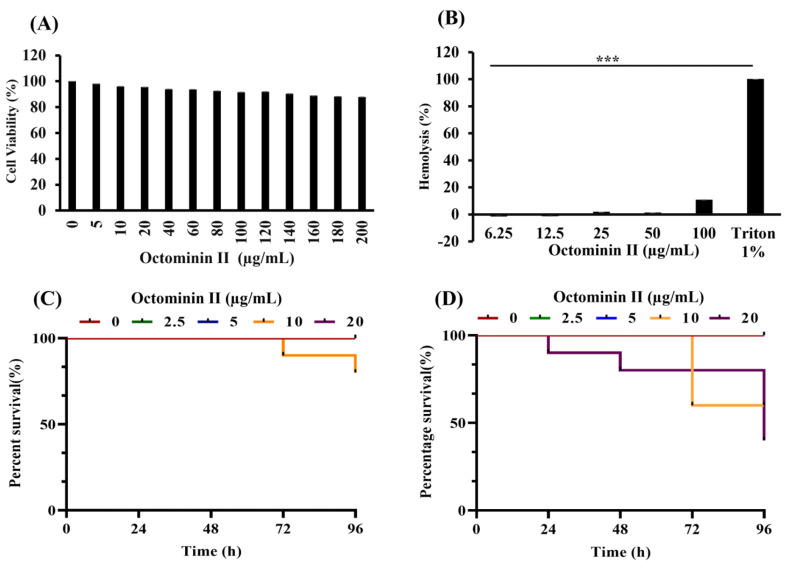
In vitro and in vivo toxicity of Octominin II. (**A**) Cytotoxicity of Octominin II. RAW 264.7 cells were treated with Octominin II (0–200 µg/mL) and then the cell viability was analyzed using the Cytox cell viability assessing kit. Bars indicate the mean ± standard deviation (*n* = 3). (**B**) Hemolysis activity of Octominin II. Mouse RBCs were treated with Octominin II (6.25–100 µg/mL) 1× PBS as the negative control, and Triton-X (10 µg/mL) as the positive control, which shows 100% hemolysis were incubated for 1 h. After that, RBCs were centrifuged, the supernatant was separated, and absorbance was measured at 490 nm. Bars indicate the mean ± standard deviation. *** *p* < 0.001 compared to the negative control (*n* = 3). (**C**) In vivo toxicity analysis of Octominin II (0–20 µg/mL) using zebrafish embryos (4 hpf). (**D**) In vivo toxicity analysis of Octominin II (0–20 µg/mL) using zebrafish larvae 72 hpf. Survival of embryos and larvae was assessed 96 h post treatment (*n* = 10).

In vivo cytotoxicity was assessed using RAW 264.7 cells treated with varying concentrations (0–200 µg/mL) of Octominin II. The results revealed that Octominin II caused very low cytotoxicity in RAW 264.7 cells, wherein the calculated IC_50_ is 341.45 µg/mL (Figure 9A).

To evaluate the hemolytic activity of Octominin II, murine red blood cells were treated with varying concentrations of Octominin II (6.25–100 µg/mL). Octominin II showed very low hemolytic activity when compared to that of the positive control (Triton X; 10 g/mL) (Figure 9B).

In vivo toxicity of Octominin II was measured by treating zebrafish embryos (4 hpf) and larvae (72 hpf) at different concentrations (0–20 µg/mL) of Octominin II and observed at 24 h intervals. Octominin II exhibits concentration-dependent toxicity in both zebrafish embryos and larvae. Considering the mortality and survival patterns, the highest concentration (20 µg/mL) showed high mortality during the observed period (96 h). (Figure 9C,D). Calculated LD_50_ values are 52.72 and 73.56 µg/mL for zebrafish embryo and larvae, respectively.

### 2.10. In Vivo Efficacy of Octominin II against C. albicans Infection in Zebrafish Model

**Figure 10:**
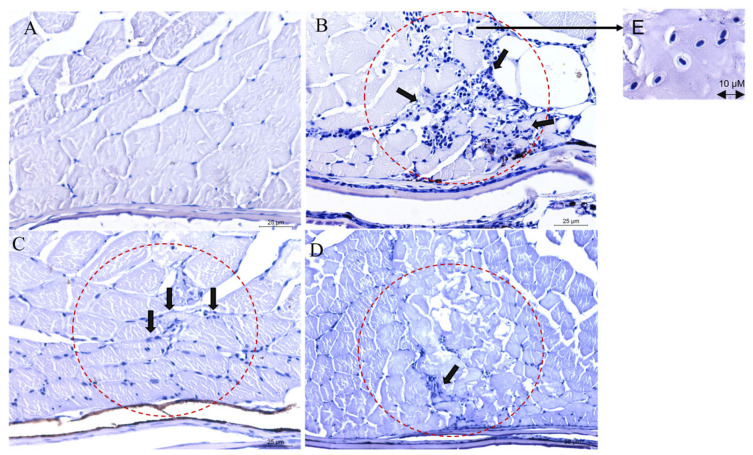
In vivo activity of Octominin II against *C. albicans*. After *C. albicans* was challenged, Octominin II was applied topically. Fish were sacrificed after 24 h, and the tissue muscles were processed and stained with PASH staining. (**A**) Negative control—with no *C. albicans* challenge (**B**) Control with *C. albicans* infection. (**C**) *C. albicans*-infected and Octominin II topically applied. (**D**) Octominin II was injected beneath the hypodermis of the dorsal muscle and topically applied. (**E**) Zebrafish neutrophil cells. Dotted circles (red) show the inflamed tissue area with accumulated neutrophils (black arrow) in respective groups.

*C. albicans*-infected zebrafish dorsal muscle tissues were investigated using PASH staining (Figure 10). *C. albicans*-challenged zebrafish showed higher neutrophil flooding than all the other groups, indicating a high level of inflammation (Figure 10B). The Octominin II-treated group showed less neutrophil infiltration, indicating low integrity of inflammation (Figure 10C). This finding suggests that Octominin II decreases the virulence of *C. albicans*, which prevents further invasion of *Candida*.

## 3. Discussion

*C. albicans* is one of the most common pathogens that cause candidiasis in immunocompromised patients [23]. The major concern in *C. albicans* infection is its acquired resistance to most existing antifungal drugs [24]. Therefore, this study aimed to develop AMPs as alternative agents to combat this opportunistic pathogen. AMPs are key elements in innate immunity that inhibit microorganisms through different mechanisms, such as membrane damage and inactivation of intracellular targets [25]. In our previous study, we confirmed that the anticandidal activity of Octominin was stronger than that of fluconazole. In this study, we modified the original Octominin to Octominin II, a shorter-length peptide (18 aa), and confirmed its anticandidal and antibiofilm activities. Peptides from various sources have shown anticandidal activity against *C. albicans.* For example, human-based peptides (LL-37, histatin 5, and lactoferrin) [26], plant-based peptides such as NaD1 from *Nicotiana alata* [27], Psd1 from *Pisum sativum* [28], marine mollusk-based peptides; Nv-p1, Nv-p2, and Nv-p3 from *Nerita versicolor* [29], and the insect peptide Gomesin, *Acanthoscurria gomesiana* [30], have been identified and characterized.

When physiochemical parameters were considered, Octominin was found to be a cationic peptide with high hydrophobicity. Cationic peptides initiate peptide–membrane binding and lyse cells via electrostatic interactions with negatively charged bacterial membranes [31]. *C. albicans* has a cell wall composed of a β-glucan-chitin skeleton [31,32], which is also negatively charged and attracts peptide binding [33]. Octominin II also exhibited a hydrophobicity index of 0.46. The antimicrobial activity of peptides can be increased by increasing their hydrophobicity to a threshold level [34]. This suggests that the cationic properties, together with the hydrophobicity of Octominin II, may affect the inhibition of *C. albicans*.

SEM confirmed that Octominin II treatment caused ultrastructural changes on the cell surface of *C. albicans*. Pores and deformities were observed in treated *C. albicans* cells, but not in untreated cells. Similar cell wall damage has been reported in other AMP-treated *C. albicans* [35,36]. The cell wall of *C. albicans* is a vital factor in host–fungus interactions leading to infection, and such defects in the cell wall can adversely affect the growth and development of *Candida* spp. [36,37]. Morphological changes caused by Octominin II can disturb the growth and development of *Candida,* which can eventually lead to growth inhibition and cell death. Based on the results of PI uptake, it was evident that Octominin II altered the membrane integrity of *C. albicans*. PI is impermeable to live cells and only penetrates dead cells. However, it is permeable through damaged cell membranes, can bind to DNA or RNA in the cell, and emits a red fluorescence signal [38]. Since higher PI uptake was observed in Octominin II-treated cells, we suggest that Octominin II damages the cell membrane, allowing PI to enter the cells and eventually contributing to the inhibition of *C. albicans.*

ROS production can be induced with antimicrobial agents, which leads to cellular stress. Numerous studies have shown peptide-mediated ROS generation connected to fungicidal activity [39,40,41]. Octominin II increased ROS levels in *C. albicans*, and ROS accumulation was associated with its inhibitory activity. With ROS production, mitochondria can also be negatively affected and cellular ATP production can be halted, which may indirectly trigger an inhibitory effect in *C. albicans* [42].

The DNA-binding ability of Octominin II was evaluated by observing the retardation of DNA movement after treatment with Octominin II. Positively charged peptides and negatively charged DNA can be attracted, which may lead to the accumulation of peptides in cells, wherein DNA and RNA may be the intracellular targets of peptides [43]. Previous studies have examined the DNA-binding ability of different peptides, including Buforin II [44], NK-18 [45], LL-37 [46], CecXJ-37N [47], and Octoprohibitin [48]. When peptides bind to DNA, they can disturb cellular metabolic activities, such as transcription and protein synthesis, thus leading to cell death.

To investigate the effects of Octominin II at the molecular level, we examined the translational responses of selected genes from *C. albicans*. *CDR1* is a prominent multidrug transporter, and its high expression levels render *C. albicans* resistant to drugs via ATP-binding cassette transporters. To block the activity of this gene, a theoretical approach is to reduce the intracellular ATP levels, which has not yet been clinically practiced [49]. Tanida et al. [49] reported that AMPs (Pep2, HNP1, and Hst5) inhibit *C. albicans* by promoting ATP efflux, whereas AMP treatment lowers *CDR1* and *CDR2* expression. Tup1 is a repressor of the yeast-to-filamentous transition process, supporting hyphae formation [50]. It was also found that Tup1 is associated with the transcription of several virulence genes, such as *ALS3* and *ECE1* [51]. *AGE3* is also associated with drug resistance; fluconazole-resistant clinical *C. albicans* isolates acquire fluconazole sensitivity after deleting the *AGE3* gene [51]. *GSC1* is associated with echinocandin resistance and is one of the available drug groups for the treatment of *C. albicans* infections in humans [52,53]. Secreted aspartic proteases (Saps) are a family of the most studied virulence factors of *C. albicans* and are one of the major hydrolytic enzymes secreted by *C. albicans* [54]. *SAP2* and *SAP9* are known to degrade host proteins, promote host tissue invasion, and confer higher virulence in *C. albicans* [54]. Interestingly, Octominin II suppressed the mRNA levels of all the selected genes in this study, demonstrating its potential for use in clinical trials. Although the mechanism associated with transcriptional suppression has not been clearly identified, it can be attributed to the interference of Octominin II with genomic DNA and RNA. This could be attributed to the induction of ROS production by Octominin II.

Biofilm formation by *C. albicans* is considered as an essential factor in its pathogenicity and facilitates drug resistance. *C. albicans* forms complex biofilm structures that exacerbate health-associated complications, such as fatal candidemia, and escalate health-related costs due to additional medications and device renewal [55,56]. Octominin II exhibited concentration-dependent antibiofilm activity. The results of this study confirmed that Octominin II can inhibit biofilm formation and eradicate preformed biofilms. Further studies are needed to identify the exact mechanism underlying the antibiofilm activity. Moreover, the use of Octominin II as a coating for medical devices can also be tested, which would reduce biofilm formation and eventually reduce the health and economic burden caused by *Candida* spp.

Neutrophils are the first line of innate immune defense against several fungal pathogens, including *Candida* spp. Patients with acquired or inherited neutrophil defects are more likely to develop invasive candidiasis [57]. Several studies have identified the role and importance of neutrophils in *Candida* infection by studying protein defects such as CAARD9 deficiency, a rare autosomal recessive primary immunodeficiency disorder [57]. The virulence of *C. albicans* was altered using cold atmospheric plasma, and the results revealed reduced neutrophil migration and inflammation in the vulvovaginal tissues of female mice [58]. In our study, the Octominin II-treated group had less neutrophil flooding, suggesting a possible reduction in the virulence of *C. albicans* when compared to that of the *C. albicans*-challenged group incorporated with Octominin II. Transcriptional analysis also suggests that the virulence and pathogenicity of *C. albicans* can be suppressed by Octominin II, which may lead to defects in the invasion and infection of *C. albicans* into the host tissue.

In this study, the ability of Octominin II to inhibit *Candida* was evaluated, and Octominin II was found to inhibit *C. albicans* via different mechanisms. Octominin II damages and alters the integrity of yeast cells, thereby increasing ROS production. In addition, it damages DNA and RNA, which may lead to defects in transcriptional regulation and affect the survival mechanisms of *C. albicans.* Molecular analysis of gene expression revealed that Octominin II suppresses the virulence and pathogenicity of *C. albicans.* Taken together, Octominin II has the potential to be used against *C. albicans* to mitigate the burden of *Candida* infections in the future.

## 4. Materials and Methods

### 4.1. Designing and Synthesis of the Octominin II

Octominin II (18 AAs) was designed by removing five amino acids from the C-terminus of the Octominin sequence. A three-dimensional image was generated using PEPstrMOD, choosing the cluster prediction option (http://osddlinux.osdd.net/raghava/pepstrmod/index.php (accessed on 20 May 2023) and visualized using the BIOVIA Discovery Studio Visualizer (Biovia, Dassault systems, Vélizy-Villacoublay, France). The physicochemical properties of Octominin II were determined using the Peptide R package version 2.4.5 [59]. A helical wheel was drawn using the NetWheels online tool (http://lbqp.unb.br/NetWheels/ (accessed on 20 May 2023). Octominin II was synthesized using a solid-phase peptide synthesis method (AnyGen, Gwangju, Korea) and purified by reverse-phase high-performance liquid chromatography. The peptide was dissolved in nuclease-free water (1 mg/mL) and stored at −20 °C.

### 4.2. Determination of Minimum Inhibitory Concentration (MIC), Minimum Fungicidal Concentration (MFC), Time-Kill Kinetics, and pH Dependent Inhibitory Activity of Octominin II

Initially, the antimicrobial activity of Octominin II was screened against three *Candida* spp., *C. albicans*, *C. auris*, and *C. glabrata*. MIC was determined using the broth microdilution method described in our previous study [48]. *C. albicans*, *C. auris*, and *C. glabrata* were grown in PDB or PDA. All three *Candida* species (fungal cell suspension of 1 × 10^6^ CFU/mL) were added to a 96-well plate and treated with Octominin II (0–250 µg/mL) in total volume of 200 µL. The plates were incubated at 37 °C for 24 h, and the optical density was measured at 590 nm. The MIC was determined as the Octominin II concentration at which zero visual fungal growth (no optical density when compared with the negative control) was observed. The MBC was determined using the agar plating method by plating Octominin II-treated *C. albicans* (≥MIC). Time-kill kinetics were determined by measuring the optical density of Octominin II-treated *C. albicans* (1 × 10^6^ CFU/mL) at 3 h intervals.

The pH-dependent activity of Octominin II was also tested. A pH gradient was prepared from pH values of 3, 4, 5, 5.5, and 7 using PDA. An overnight culture of *C. albicans* was prepared, and 10% inoculum was added to fresh PDB. Cells were allowed to grow until they reached a density of 1 × 10^6^ CFU/mL. After that, the cell suspension was added to a 96-well plate and treated with varying concentrations (30, 50, 80, 100, 120, and 150 µg/mL) of Octominin II and incubated at 37 °C for 24 h. The percentage inhibition was calculated and compared with the negative control.

### 4.3. FE-SEM for Morphological and Structural Analysis of Octominin II Treated C. albicans

FE-SEM analysis was performed to determine the morphological and structural changes in Octominin II-treated *C. albicans.* Octominin II treatments were performed at MIC (80 µg/mL), MFC (120 µg/mL), and fluconazole (280 µg/mL) with *C. albicans* (1 × 10^6^ CFU/mL) and incubated at 37 °C for 10 h. Fungal cells were collected by centrifugation at 1500× *g* for 10 min. The cell pellets were washed twice with 1× PBS, suspended in 1× PBS, and pre-fixed with 2.5% glutaraldehyde for 30 min. Then, the pre-fixed cells were obtained by centrifuging again at 1500× *g* for 10 min, washed with 1× PBS, and then dehydrated using serial ethanol concentrations (30, 50, 70, 80, 90, and 100%). The fixed cells were dried and coated with platinum by ion sputtering (E–1030, Hitachi, Tokyo, Japan). Finally, the cells were observed by field-emission scanning electron microscopy (FE-SEM; Sirion FEI, Eindhoven, The Netherlands).

### 4.4. Propidium Iodide Uptake Assay (PI/FDA) and Reactive Oxygen Species (ROS) Generation

A PI uptake assay with fluorescein diacetate (FDA) staining was performed to identify any effect of Octominin II on membrane permeability or cell membrane integrity. Propidium iodide (PI)/FDA staining was performed according to the method described by Jayathilaka et al., 2021 [20]. Briefly, *C. albicans* (1 × 10^6^ CFU/mL) was treated with Octominin II with the MIC (80 µg/mL) and MFC (120 µg/mL) as the positive control (fluconazole, 280 µg/mL). Samples were incubated at 37 °C for 10 h, and then the cells were collected by centrifugation at 1500× *g* for 10 min. The cell pellet was washed with 1× PBS and resuspended in 1 mL PBS. For the PI uptake assay, 50 µg/mL of PI (Sigma Aldrich, Saint Louis, MO, USA) and 40 µg/mL FDA (Sigma Aldrich, Saint Louis, MO, USA) were added and incubated for 30 min at room temperature (28 °C) in the dark. Then, the excess stain was removed using 1× PBS, and cells were resuspended in 1× PBS (50–100 µL), and cell suspension (5 µL) was added onto a glass slide and observed under confocal laser scanning microscopy (CLSM). Red fluorescence was measured at excitation and emission wavelengths of 535 and 617 nm, respectively, and green fluorescence was measured at excitation and emission wavelengths of 488 and 535 nm, respectively.

ROS production in *C. albicans* was assessed using the 2′,7′-dichlorodihydrofluorescein diacetate (H_2_DCFDA) assay. After treatment and collection of *C. albicans* as described above, *C. albicans* cells were washed twice with 1× PBS and resuspended in 1 mL of 1× PBS. H_2_DCFDA staining was performed by adding 50 µg/mL H_2_DCFDA (Invitrogen, Carlsbad, CA, USA) to the cell suspension, followed by incubation for 30 min at room temperature in the dark. Excess H_2_DCFDA was washed with 1× PBS and resuspended in 50–100 µL of 1× PBS, and 5 µL of the cell suspension was added to a glass slide and observed under confocal laser scanning microscopy (CLSM). Excitation and emission wavelengths for green fluorescence were 488 and 535 nm, respectively. For H_2_DCFDA staining to determine ROS generation, 50 µg/mL H_2_DCFDA (Invitrogen, Carlsbad, CA, USA) was added and incubated for 30 min at room temperature in the dark. Excess H_2_DCF-DA was washed with 1× PBS and resuspended in 50–100 µL of 1× PBS, and 5 µL of the cell suspension was added to a glass slide and observed under confocal laser scanning microscopy (CLSM). Excitation and emission wavelengths for green fluorescence were 488 and 535 nm, respectively.

### 4.5. DNA and RNA Mobility Shift Assay

DNA and RNA mobility shift assays were performed to determine whether Octominin II binds to the nucleic acids of *C. albicans*. First, the genomic DNA of *C. albicans* was isolated by the method described by [60], and RNA was isolated by NucleoSpin^®^ RNA isolation kit according to the manufacturer’s protocol (NucleoSpin, Mcherey-Nagel, Düren, Germany). Isolated genomic DNA and RNA were treated with different weight ratios of Octominin II (1:0, 1:1, 1:2, 1:4, and 1:8) in a 20 µL reaction mixture and incubated at 37 °C for 30 min. Then, the total volume of the DNA or RNA with Octominin II was loaded onto 0.8% agarose gel. The RNA Octominin II mixture was loaded onto 1% gel and electrophoresed using an electrophoresis system (Mupid-2plus, TaKaRa, Tokyo, Japan). The gel was visualized using a gel imaging system (MaXidoc G2; DAIHAN, Wonju, Korea).

### 4.6. Transcriptional Analysis of C. albicans after Octominin II Treatment

The gene expression profiles of the selected genes of *C. albicans* were analyzed following Octominin II treatment (Table 2). *C. albicans* (1 × 10^6^ cells) was treated with Octominin II with 0.5 MIC and MIC concentrations with the positive control (fluconazole 280 µg/mL) and incubated at 37 °C for 3 h. Then, the cell pellet was obtained by centrifugation at 1500× *g* for 10 min at 4 °C. PBS washed cell pellet was used to isolate total RNA by NucleoSpin^®^ RNA isolation kit, according to the manufacturer’s protocol (740955.05, NucleoSpin, Mcherey-Nagel, Düren, Germany). RNA concentration and purity were determined using a NanoDrop spectrophotometer (Thermo Fisher Scientific, Waltham, MA, USA), and cDNA was synthesized using the Prime Script 1st strand cDNA Synthesis Kit (TaKaRa, Tokyo, Japan), according to the manufacturer’s protocols. Quantitative real-time polymerase chain reaction (qRT-PCR) was performed using the Thermal Cycler Dice Real Time System III (TaKaRa, Tokyo, Japan) with the total reaction mixture (10 µL), which included cDNA template (3 µL), 1 µL of each forward and reverse primers (10 µM each), and 5 µL of TB green Premix Ex Taq II (TaKaRa, Tokyo, Japan). The three-step PCR was performed with a single dissociation step. The relative mRNA expression fold values were calculated following the 2^−ΔΔCT^ method, wherein the reference control gene was Actin 1 [61].

### 4.7. Antibiofilm Activity Assays

*C. albicans* biofilms were prepared for CLSM, according to a previously described method with minor modifications [62]. For the CLSM study, *C. albicans* was grown to 10^6^ CFU/mL in PDB, and desired amounts of *C. albicans* culture and Octominin II were added to Falcon^®^ 96-well Black/Clear Flat Bottom plates (353219, Corning, NY, USA) up to a total volume of 100 µL and incubated at 37 °C for 24 h for biofilm formation. The biofilms were carefully washed twice with 1× PBS to remove planktonic cells. The biofilms were stained with 10 mM SYTO9 (Thermo Fisher Scientific, Waltham, MA, USA) and kept in the dark for 30 min. Subsequently, the remaining dye was washed off and observed using a CLSM with Z-stacking (Zeiss LSM 880 with Airyscan, Zeiss, Oberkochen, Germany). Plates were visualized at a wavelength of 488 nm. Quantification of the formed biofilms was performed using COMSTAT software [63], where the biofilm biomass (µm^3^/µm^2^) and the biofilm thickness were quantified.

For the biofilm inhibition assay, *C. albicans* culture with the desired amount of Octominin II was added to a 96-well plate until a final volume of 100 µL and incubated at 37 °C for 24 h. For biofilm eradication assay, 100 µL of *C. albicans* culture was added to the 96-well plate and incubated at 37 °C for 48 h. The biofilm formed was washed twice with 1× PBS after removing the medium. Next, formed biofilms were treated with 50, 80, 100, and 120 µg/mL of Octominin II, and fluconazole (280 µg/mL) and new media were added until a final volume of 100 µL and incubated at 37 °C for 24 h.

A crystal violet assay was performed to measure the remaining biomass of *C. albicans* biofilms after Octominin II treatment. First, the remaining culture medium was carefully removed, and the biofilm-containing wells were washed twice with 1× PBS to remove non-attached cells without disturbing the remaining biofilm. Then, crystal violet (0.1%) was added to each well and incubated for 30 min at room temperature. After that, the plate was washed with distilled water, and then 100 µL of ethanol was added to each well and kept for 10 min. Finally, the plate was read at 595 nm using a microplate reader.

The biofilm formation inhibition percentage was calculated using the following equation:

Formed biofilm biomass (%) = [(A595 of Treatment)/(A595 of Negative control)] × 100%

Biofilm formation inhibition (%) = 100 − [(A595 of Treatment)/(A595 of Negative control)] × 100%

The remaining biofilm upon Octominin II treatment was calculated using the following equation:

Remaining biofilm biomass (%) = [(A595 of Treatment)/(A595 of Negative control)] × 100%

Biofilm eradication (%) = 100 − [(A595 of Treatment)/(A595 of Negative control)] × 100%

### 4.8. In Vitro and In Vivo Toxicity Analysis and Hemolysis Activity of Octominin II

The in vitro cytotoxicity of Octominin II was determined in murine macrophages (RAW 264.7 cells). Cells were cultured in Dulbecco’s modified Eagle’s medium (Welgene, Gyeongsan, Korea), containing 10% (*v*/*v*) fetal bovine serum (Welgene, Gyeongsan, Korea) with an antibiotic–antimycotic solution (Thermo Fisher Scientific, Waltham, MA, USA), and incubated at 37 °C in a humidified atmosphere at 5% CO_2_ for 24 h. The cells were then seeded into a 96-well flat bottom microtiter plate at a density of 1 × 10^4^ cells per well (100 µL) and allowed to adhere by incubating at 37 °C for 24 h. After that, the medium was aspirated, and the cells were washed with sterile 1× PBS. Cells were treated with Octominin II (0–200 µg/mL), and untreated cells were used as negative controls. After incubation, cell viability was measured using the EZ-Cytox cell viability assay kit (DoGenBio Co., Seoul, Korea), according to the manufacturer’s protocol. The hemolytic activity of Octominin II was assessed using murine red blood cells (RBC), according to the method described by Jayathilaka et al. (2021), with minor modifications [48]. Briefly, the collected RBCs were washed (1× PBS) and treated with different concentrations of Octominin II (0–100 µg/mL), and 1% of Triton X-100 (Sigma Aldrich, Munich, Germany) was added as the positive control, and PBS was used as the negative control. The treated RBCs were then incubated at room temperature for 1 h. The supernatant was separated by centrifugation, and the absorbance of the supernatant was measured at 415 nm using a microplate spectrophotometer (BioRad, Saint Louis, MO, USA). Hemolytic activity was assessed as a percentage using the following equation:

Hemolysis % = [(Ab test − Ab PBS)**/**(Ab Triton − Ab PBS)] × 100%

In vivo toxicity of Octominin II was assessed in zebrafish (*Danio rerio*) embryos and larvae. Zebrafish embryos (4 hpf) and larvae (72 hpf) were placed in 96-well flat bottom microtiter plates, with one embryo/larva per well (*n* = 10). Embryos or larvae were treated with Octominin II (0–25 µg/mL) at 28 °C for 96 h, and mortality was determined.

### 4.9. In Vivo Anticanidal Activity of Octominin II against C. albicans in Zebrafish

An in vivo experiment was conducted according to the method described by Kulatunga et al. (2019) with minor modifications [64]. *C. albicans* was grown to a density of 1 × 10^9^ cells/mL. The fish were divided into three groups: (1) negative control (PBS), (2) *C. albicans* challenge (5 µL of the *C. albicans* suspension; 10^3^ cells/mL), and (3) *C. albicans* challenge with Octominin II treatment (*C. albicans* 10^3^ cells/mL and 0.005 mg of Octominin II). Zebrafish (0.4 g body weight) were anesthetized using 160 µg/mL buffered tricaine (ethyl 3-aminobenzoate methane sulfonate; Sigma-Aldrich, Saint Louis, MO, USA), and Octominin II treatments were injected into the dorsal muscle of the zebrafish and kept in 28 °C. The fish were observed for mortality and health abnormalities. Muscle samples were collected 48 and 96 h after the challenge/treatment (*n* = 3 from each group). The collected tissues were subjected to periodic acid-Schiff hematoxylin (PASH) staining for histopathological observation according to the method described by Kulatunga et al. [64].

### 4.10. Statistical Analysis

All experiments were statistically analyzed using GraphPad Prism software version 8 (GraphPad Software Inc., La Jolla, CA, USA). All in vitro and in vivo assays were performed in triplicate and analyzed using the one-way ANOVA test, while an unpaired *t*-test was used for the transcriptional analysis with significance levels of * *p* < 0.05, ** *p* < 0.01, and *** *p* < 0.001.

## 5. Conclusions

In conclusion, Octominin II inhibited *C. albicans* growth, and a positive correlation was found between the MIC and MFC of 80 and 120 µg/mL, respectively. Our results revealed that Octominin II exerts multiple modes of action to inhibit *C. albicans* such as physical cell membrane damage, increased plasma membrane permeability, induction of high levels of ROS, and binding with intracellular DNA and RNA. Moreover, Octominin II showed good antibiofilm activity by successfully inhibiting and eradicating *C. albicans*-derived biofilms. Calculated IC_50_ was 341.45 µg/mL (RAW 264.7 cells), and LD_50_ was 52.72 µg/mL (zebrafish embryos) and 73.56 µg/mL (larvae). Overall, Octominin II could be a potential therapeutic agent against the multidrug-resistant *Candida* species; however, further experiments are required to confirm the clinical application of Octominin II.

## Figures and Tables

**Table 1:** Physiochemical properties of Octominin II.

Property	Octominin (Value/Units)	Octominin II (Value/Units)	Measurement
Net charge	+5.00	+2.46	Sum of the charges of a peptide amino acid
Isoelectric point	12.48	11.66	pH value that a molecule carries no or neutral net electrical charge
Aliphatic index	114.78	134.38	Relative volume of a peptide occupied by the aliphatic side chains
Instability index	78.99	12.51	Stability of a peptide
Boman index	1.86 kcal/mol	−0.28 kcal/mol	Potential peptide-interaction of a peptide
Hydrophobicity index	0.43	0.46	Relative solubility of the peptide

**Table 2:** Gene-specific primers of *C. albicans* used in this study.

Name of the Gene	Forward Sequence (5′-3′)	Reverse Sequence (3′-5′)	Accession Number
Multidrug resistance protein-*CDR1*	ACAATACAAGACCAGCATCTCC	AGACCCATTACAAGTTGACCG	XM_718116.2
Chromatin-silencing transcriptional regulator-*TUP1*	ATACATTGTCAACCCCACCC	AGTCTTTGGAGAACGCTGG	XM_713975.2
ADP-ribosylation factor GTPase-activating, protein encoding gene/ARF-GAP-encoding gene *AGE3*	TCCATGATCCAGAAACTCGTAG	ACTCCACACATTCTAAACAAATG	XM_708684.2
Beta-1,3-glucan synthase catalytic subunit-*GSC1*	ACTGCTTACAACTCCCCAAC	CCATTCGAAAAGTGTGGCAAG	XM_716336.2
Secreted aspartyl proteinase-2-*SAP2*	CAAGGAGTCATTGCTAAGAATGC	AGCATTATCAACCCCACCG	XM_705955.2
Secreted aspartyl proteinase-9-*SAP9*	CATCTTCATCTGGCACCTCTAC	CGAAAGCAACAACCCATACAC	XM_707636.2
*Actin 1*	TGAAGCCCAATCCAAAAGAGG	TTTCCATATCGTCCCAGTTGG	XM_019475182.1

## Data Availability

Available upon request.

## Supplementary Materials

The following supporting information can be downloaded at: https://www.mdpi.com/article/10.3390/ijms241814053/s1.
