# Supplementary material for: Antifungal Efficacy of Antimicrobial Peptide Octominin II against Candida albicans
Source: Int J Mol Sci. 2023 Sep 13;24(18):14053. doi: 10.3390/ijms241814053 (PMC10531694; doi:10.3390/ijms241814053)

Supplementary Figure S1. Purity and molecular weight of Octominin.

II. Chromatogram

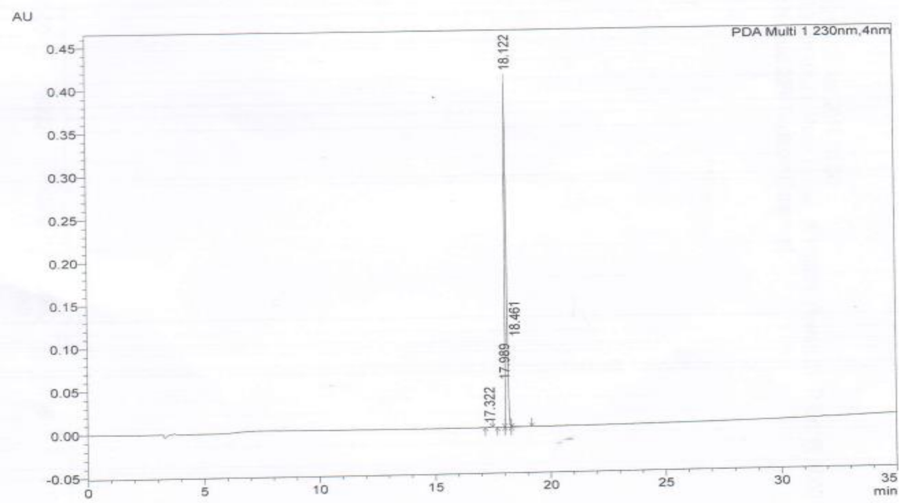

<Peak Table>

PDA Ch1 230nm

Peak#	Ret. Time	Area	Area%
1	17.322	4237	0.202
2	17.989	3295	0.157
3	18.122	2064981	98.694
4	18.461	19796	0.946
Total		2092308	100.000

Molecular weight (Octominin II)

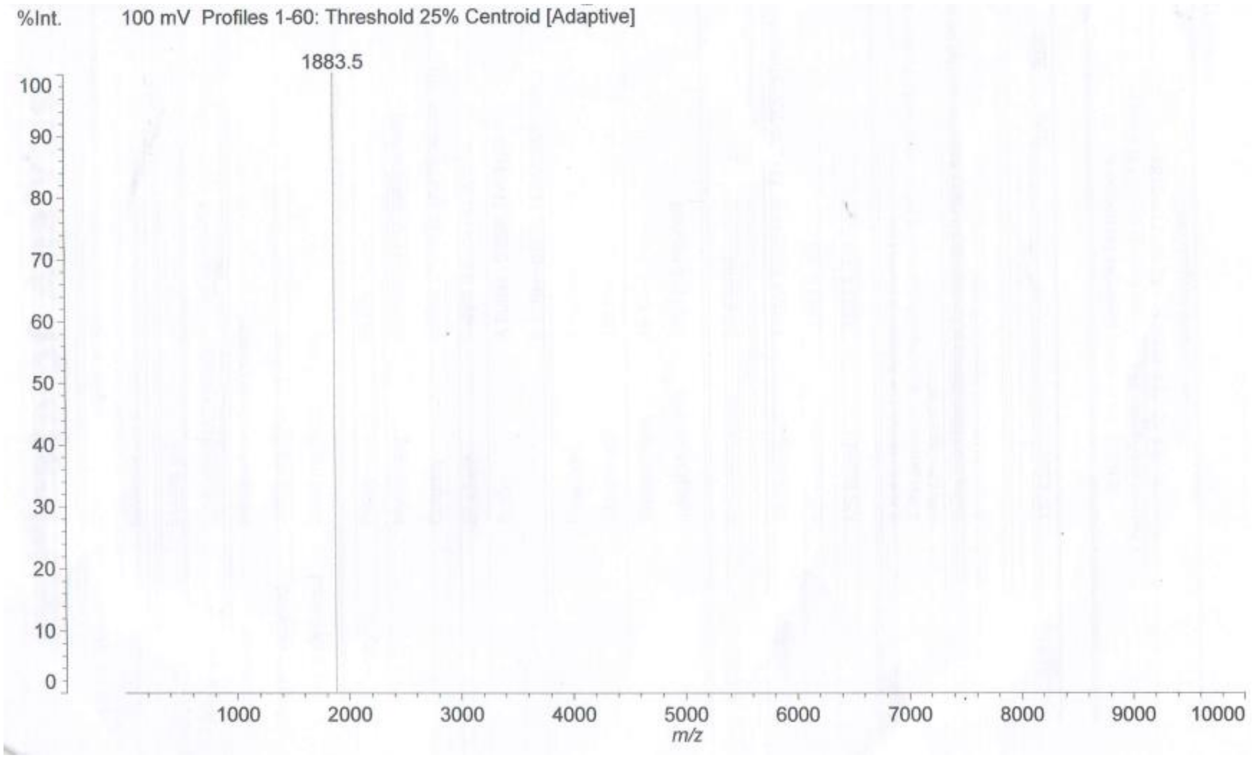

Supplement: Supplementary file 1 [file ijms-24-14053-s001.zip › ijms-2560653-supplementary.pdf]
